# Good results at 2-year follow-up of a custom-made triflange acetabular component for large acetabular defects and pelvic discontinuity: a prospective case series of 50 hips

**DOI:** 10.1080/17453674.2021.1885254

**Published:** 2021-02-15

**Authors:** Marieke Scharff-Baauw, Miranda L Van Hooff, Gijs G Van Hellemondt, Paul C Jutte, Sjoerd K Bulstra, Maarten Spruit

**Affiliations:** a Orthopaedic Department, Sint Maartenskliniek, Nijmegen;; b Orthopaedic Department, University Medical Centre Groningen;; c Orthopaedic Department, Medisch Centrum Leeuwarden, The Netherlands

## Abstract

Background and purpose — Custom triflange acetabular components (CTACs) are suggested as good solutions for large acetabular defects in revision total hip arthroplasty. However, high complication rates have been reported and most studies are of limited quality. This prospective study evaluates the performance of a CTAC in patients with large acetabular defects including pelvic discontinuity.

Patients and methods — Prospectively collected data of 49 consecutive patients (50 hips), who underwent an acetabular revision with a CTAC were analyzed. Follow-up (FU) was 2 years. The median age of the patients was 68 years (41–89) and 41 were women. Primary outcomes were re-revision of the CTAC and differences between the modified Oxford Hip Score (mOHS) preoperatively and at 2-year follow-up. Secondary outcomes included several patient-reported outcomes (PROMs), radiological results, complications, and a comparison between hips with and without pelvic discontinuity (PD).

Results — 1 patient (1 hip) was lost to the 2-year FU. No CTAC needed re-revision. The preoperative and 2-year FU mOHS were available in 40 hips and improved statistically significantly. All of the other secondary outcomes improved over time. 5 hips (of 45 with radiological 2-year FU) had loosening of screws. 8 hips had complications, including 3 persistent wound leakage, 3 pelvic fractures, and 1 dislocation. The mOHS and complication rate were similar in hips with and without PD.

Interpretation — Reconstruction of large acetabular defects with and without PD with this CTAC showed good improvement in patient-reported daily functioning, high patient-reported satisfaction, few complications, and no re-revisions at 2-year FU.

Acetabular revision is challenging when facing severe host bone loss and poor remaining bone quality. Pelvic discontinuity (PD) increases the difficulty of reconstructing such defects.

Custom triflange acetabular components (CTATCs) have been repeatedly suggested as good solutions to deal with large acetabular defects, even when PD is present (Sheth et al. 2013, Baauw et al. 2016, De Martino et al. 2019, Szczepanski et al. 2019, Volpin et al. 2019, Chiarlone et al. 2020, Malahias et al. 2020). A proposed advantage is the ability to customize and individualize the implant to the defect in each individual case (Berasi et al. 2015). As such, an immediately stable initial implant fixation might be accomplished. This might be due to restoring anatomical dimensions and re-distributing load anatomically, choosing the optimal center of rotation, and supporting host bone contact and osseointegration. We feel that good design of the CTAC prior to surgery, trying to achieve implant support and fixation to the best host bone quality, is important as the implant cannot be modified intraoperatively.

A disadvantage of the use of CTACs is the reported high complication rate in terms of reoperation, infection, nerve damage, and especially dislocation (Volpin et al. 2019, Chiarlone et al. 2020, Malahias et al. 2020). However, these higher rates may relate to the difficulty of revisions and severity of the acetabular bone defects encountered when using CTACs (De Martino et al. 2019, Volpin et al. 2019). As might be expected, the risk of postoperative hip dislocation is increased in these complex cases with multiple previous surgeries, extensile approaches, pre-existent leg-length discrepancies, and frequently abductor weakness (De Martino et al. 2019). An option to reduce dislocation in revision total hip arthroplasty (THA) is by using a dual mobility design (Faldini et al. 2018) and its implementation has been recommended in acetabular revision with CTACs (De Martino et al. 2019, Malahias et al. 2020).

The use of CTACs remains controversial as many studies that evaluate the performance of these implants are retrospective small case series and as such of limited quality. There is a need for prospective studies with consistent reporting of clinical, radiological, and patient-reported outcomes.

This prospective single-center study evaluates the revision rate, patient-reported outcomes, complications, and postoperative radiographs in a consecutive series of patients with large acetabular defects treated with a CTAC in which either a dual mobility cup or a constrained liner was cemented.

## Patients and methods

Prospectively collected data (questionnaires) of 49 consecutive patients (50 hips) was extracted and anonymized from the institution’s THA revision database. Inclusion criteria were an acetabular revision with a custom-made acetabular revision system (Materialise, Leuven, Belgium) and a minimum of 2 years’ follow-up. The study complied with the STROBE guidelines (von Elm et al. 2008).

The indication for the CTAC was the presence of a Paprosky type 3B acetabular defect (Paprosky et al. 1994) with or without PD in a patient for whom other options with off-the-shelf implants were not thought feasible.

### Surgery

**Figure 2.**
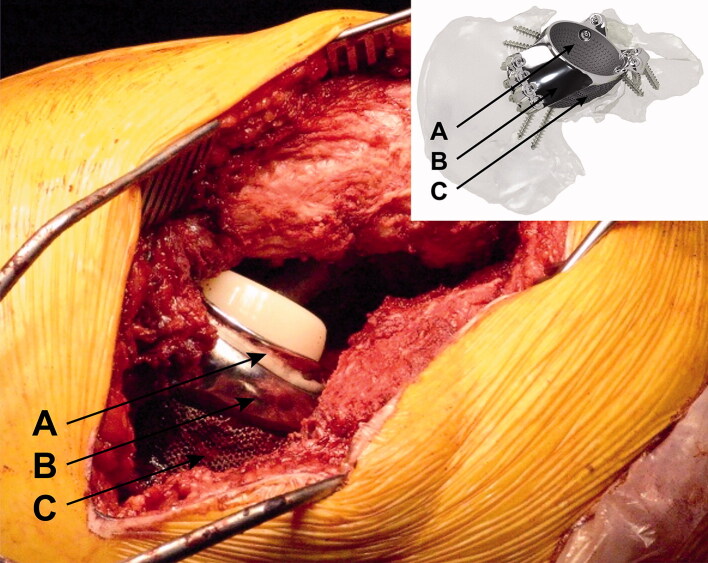
Dual mobility cup cemented into the custom-made implant. A = (place of) dual mobility cup. B = triflange cage. C = porous metal augment.

Patients were operated on between February 2013 and September 2017. A preoperative CT scan was performed for defect analyses and reconstruction planning. The surgeons gave feedback on the defect analyses and the implant orientation, determining optimal anteversion, inclination, and center of rotation of the implant. Based on this information and feedback a porous metal augment and a triflange cage, with flanges on ilium, ischium, and pubis, were designed as a monoblock, with screw fixation planned into the best host bone quality (Figure 1). All patients were operated on by an orthopedic surgeon and either another orthopedic surgeon, a fellow, or a final-year resident. A posterolateral approach was used in all patients and surgeons had a printed hemi-pelvis, trial implants, and drill guides at their disposal during surgery. Allograft was used in case of voids and/or cavitary defects between host bone and implant. Taking into account the quality of the host bone, the implant was fixed with pre-planned trajectory screws using the patient-specific drill guides. Within the implant either a dual mobility cup (48 hips) or, in the case of abductor deficiency, a constrained liner (2 hips) was cemented in the same orientation as the implant (Figure 2). Further details concerning the acetabular defect analyses and the surgical technique have previously been described (Baauw et al. 2015, 2017). Postoperatively, patients were allowed 50% weight-bearing on the operated leg for the first 6 weeks. Systemic antibiotics were routinely used perioperatively and until results of intraoperative cultures were known and low-molecular-weight heparin (LMWH) was administered in the first 6 weeks postoperatively.

Figure 1.Planning of case 17 with (A) the ultimate acetabular bone defect after subtracting all parts of the existing reconstruction and (B) the expected postoperative situation with the complete construct.

**Figure:**
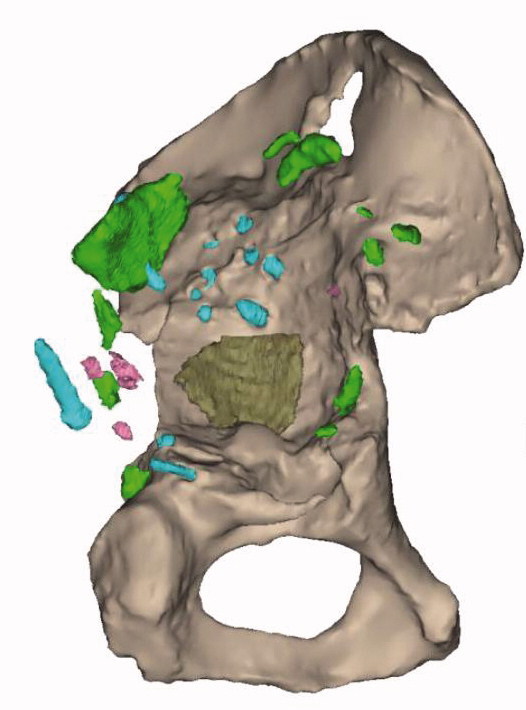

**Figure:**
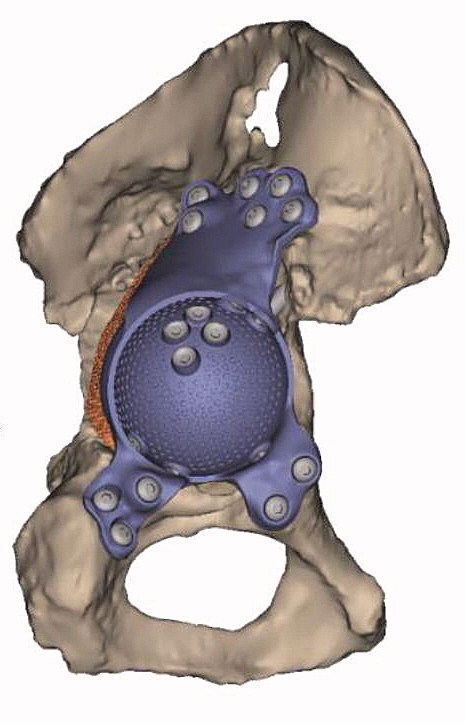

### Patients

Of the 49 included patients (50 hips), 41 were women. At the time of the hip revision surgery the median (range) age of the patients was 68 years (41–89) and their median (range) BMI was 27 (19–44). The ASA classification was 2 in most patients (30/50). The primary diagnosis was osteoarthritis (OA) in 26 patients, 41 were revised due to aseptic loosening, and the median (range) number of previous revisions was 2 (1–9). Based on preoperative analysis pelvic discontinuity (PD) was found in 16 hips. In 11 hips the stem was revised at the same time and bone graft was used in 32 hips. 2 patients (case 21 and 48) received a constrained liner instead of a dual mobility because of hip abductor deficiency. The median (range) time that patients stayed in hospital was 8 (4–28) days (Table 1, see Supplementary data).

**Table 2.** Patient-reported outcomes in medians (ranges)

	n	Preoperative score	n	2-year FU score
EQ5D-3L utility	44	0.23 (– 0.13 to 0.89)	47	0.77 (–0.20 to 1)
EQ5D-3L NRS	43	50 (7–100)	44	70 (40–100)
VASrest	45	31 (0–100)	46	2 (0–100)
VASactivity	45	78 (0–100)	46	11.5 (0–100)

EQ5D-3L, EuroQol 5 dimensions 3 level, range –0.329 to 1.

NRS, numeric rating scale, range 0–100.

VAS, visual analog scale, range 0–100.

### Primary and secondary outcomes

Our primary outcomes were re-revision of the CTAC at 2-year FU and the change in daily functioning as experienced by patients. To measure daily functioning the patient-reported modified Oxford Hip Score (mOHS) was used (Gosens et al. 2005). The preoperative mOHS (70–14) was compared with the mOHS at 2-year FU and its clinical relevance was analyzed. At 2-year FU we also looked at the mean mOHS of all available patients, including those who did not complete the mOHS preoperatively.

Secondary clinical outcomes included a comparison between preoperative and 2-year FU values of the EuroQol 5 dimensions 3 level (EQ5D-3L) utility (-0.329–1), the EQ5D-3L numeric rating scale (NRS) from 0–100 (EuroQol group 1990), and the visual analogue scale (VAS) for pain at rest and during activities (0–100). At 2-year FU the following additional clinical outcomes were measured: satisfaction with surgical result using VAS (0–100) and several core questions, which could be answered “yes” or “no.”

Complications were registered during admission and until 2-year FU and all types of complications were registered. Anteroposterior (AP) radiographs were taken at 1-year FU and 2-year FU. These were reviewed by MSB and MS for: notable breakage of the component, screw loosening (defined by radiolucency around the screws) or breakage, and bony fractures.

Finally, to explore and indicate the potential influence of PD, the re-revision rate, mOHS, and the complications in cases with PD were compared with cases without PD.

### Statistics

The primary outcome, the mOHS, was descriptively summarized, using medians and ranges, and non-parametrically tested with the Wilcoxon signed-rank test to evaluate clinical performance preoperatively versus the performance at 2-year FU. Clinical relevance of the change in mOHS was assessed using a distribution-based approach. This was calculated by taking 0.5 SD of the mean difference between the preoperative scores and the scores at 2-year FU. To further substantiate clinical relevance, the effect size was determined using Cohen’s d, which is calculated by dividing the difference in scores from preoperative to 2-year FU by the SD of the preoperative scores (Norman et al. 2003, Copay et al. 2007). An effect size of 0.2 was considered small, 0.5 moderate, and 0.8 large (Cohen 1992). The secondary clinical outcome data was descriptively summarized using medians and ranges. Missing cases for the primary outcome, the mOHS, were compared to complete cases on baseline characteristics (age, sex, BMI, primary diagnosis, number of previous revisions, stem revision, and use of bone graft and presence of PD) using the Wilcoxon signed-rank test for continuous data and Fisher’s exact test for categorical data. Statistical analyses were performed using STATA (version 13.1 for Windows; StataCorp, College Station, TX, USA). Statistical significance was defined as p < 0.05.

### Ethics, funding, and potential conflict of interests

Ethical approval from the Institutional review board was not required, as the Dutch Act on Medical Research involving Human Subjects does not apply to screening questionnaires that are part of routine clinical practice. For this study, patient data were obtained as a part of routine outcome monitoring for use in daily practice. All data were anonymized and identified for analyses and report.

Personal fees were received for faculty work from Materialise by MSB, GGvH, and MS, from Smith & Nephew by GGvH, from Zimmer Biomet by GGvH, and from DePuy Synthes by MS. SKB is the president of the Dutch Orthopedic Society and MS is chairman of the AOTK Spine.

## Results

### Primary outcomes

1 patient (1 hip) was lost to the 2-year FU (case 49) and did not respond to questionnaires or follow-up appointments due to her comorbidities. None of the remaining 49 CTACs needed re-revision at 2-year FU. The mOHS was missing in 7 cases at preoperative assessment (cases 10, 18, 24, 37, 38, 39, 50) and in 3 cases at 2-year FU (cases 21, 25, 49). In the remaining 39 patients (40 hips) with complete mOHS a statistically significant improvement was shown from 51 (24–67) to 28.5 (14–56) at the 2-year FU. The clinically relevant difference (0.5 SD) was 5 points and present in 37 out of 40 patients with complete mOHS. The effect size was large (d = 1.6). The mOHS of all available patients (n = 47) at 2-year FU, irrespective of (in-)complete baseline mOHS, was 29 (14–56).

Patients who had incomplete data for the mOHS differed statistically significantly from patients with complete data with regard to the number of previous revisions: 3.5 (1–9) previous revisions in patients with incomplete mOHS and 2 (1–9) in patients with complete mOHS. No other significant differences in baseline characteristics were shown between complete and incomplete cases,.

### Patient-reported clinical results

Our secondary outcome measures on EQ5D-3L utility, EQ5D-3L NRS, VASrest, and VASactivity improved between baseline and 2-year FU (Table 2). For these values we had 41/400 (10%) missing values.

Satisfaction with the surgical result was reported in 45 cases and was 96 (0–100). The results of the core questions are described in Table 3.

**Table 3.** Core questions at 2-year follow-up

Core question (n = 47)	Yes
Has the operation improved the mobility or function of the hip?	38
Has the pain in/around the hip lessened since the operation?	45
Are you satisfied with the results of the operation?	42
Would you recommend the operation to a family member or friend?	47

### Radiological results

AP radiographs were available of 49 hips at 1-year FU and of 45 hips at 2-year FU (Figure 3). 5 hips had loosening of screws at 1-year FU with no signs of progression at 2-year FU (cases 10, 31, 32, 38, and 42). In all of these patients screw loosening was found in 1 or more ischium screws and in one of these hips there was also screw loosening of a pubis screw (case 10) (Figure 4). The missing 4 hips at 2-year FU (case 16, 41, 43, and 50) did not show any complications at 1-year FU.

Figure 3.Case 17 (A) preoperatively and (B) at 2-year follow-up.

**Figure:**
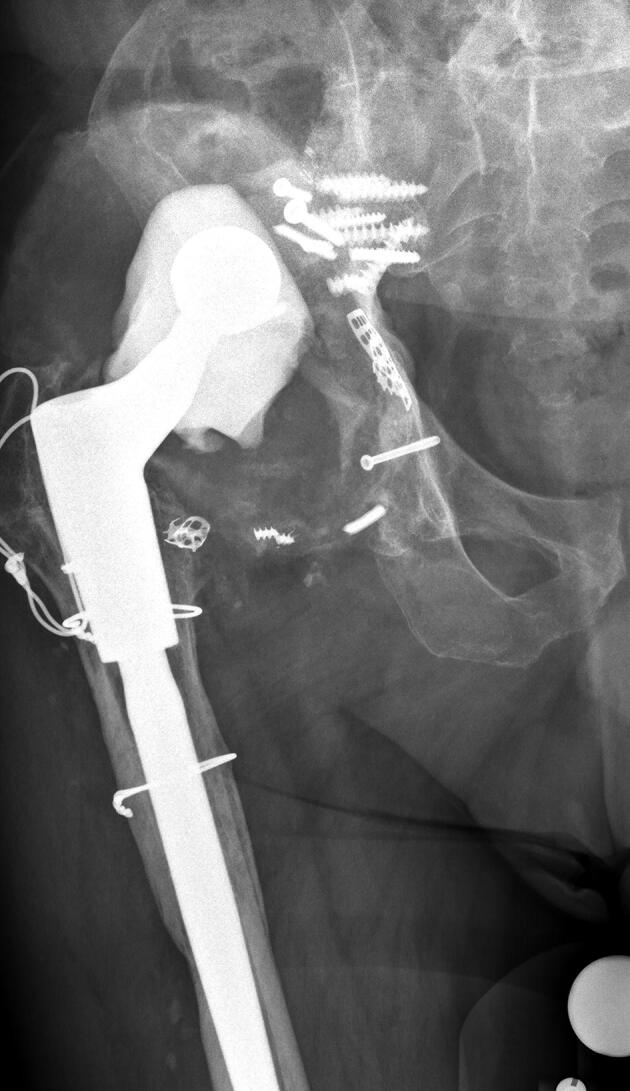

**Figure:**
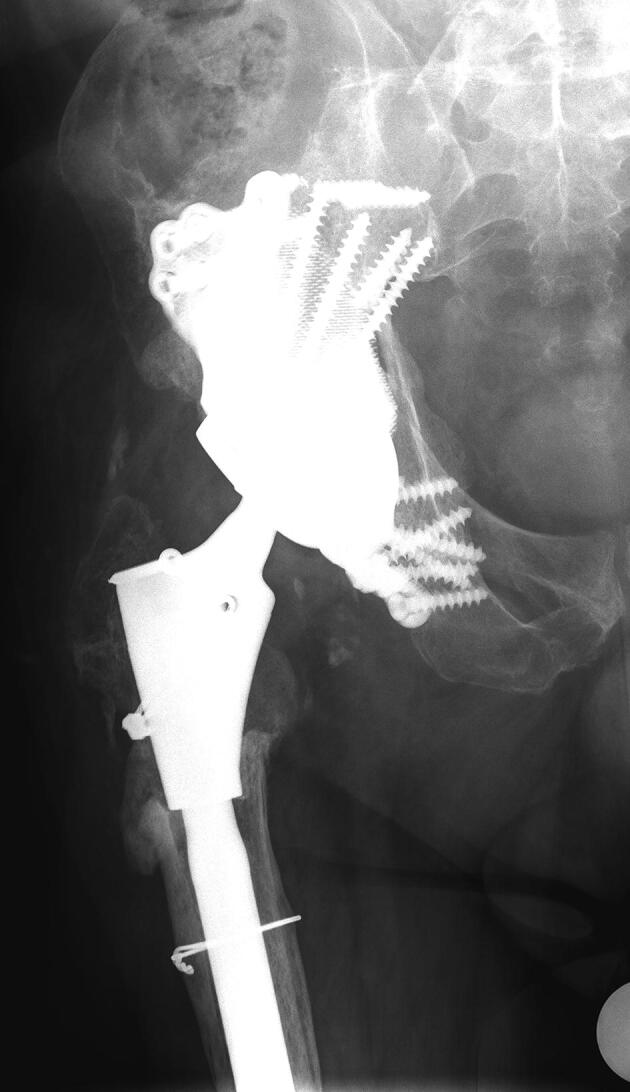

Figure 4.Case 10 (A) preoperatively, (B) at 1-year follow-up, and (C) at 2-year follow-up.

**Figure:**
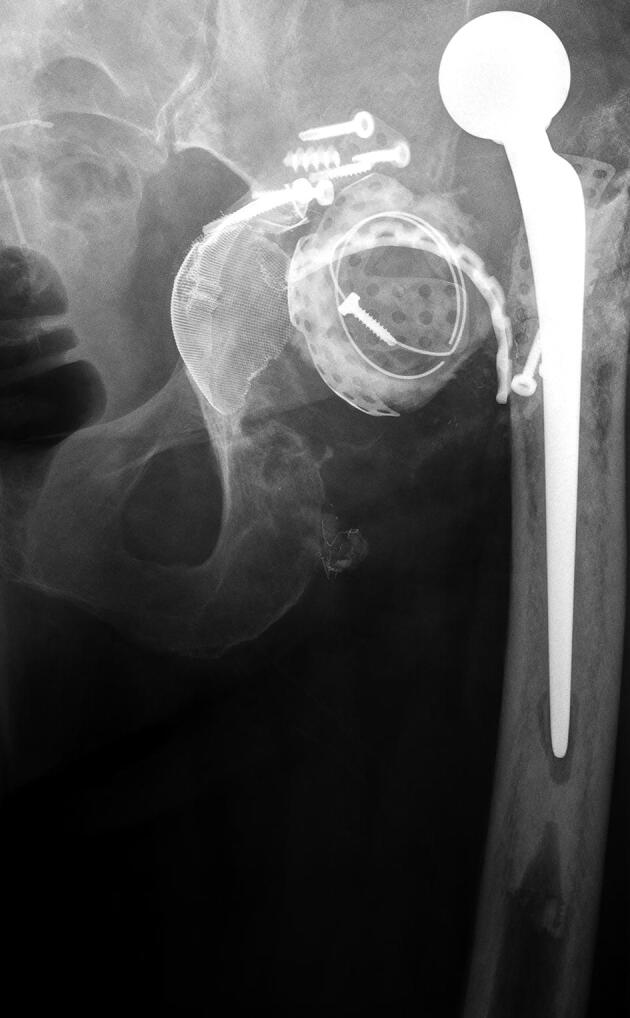

**Figure:**
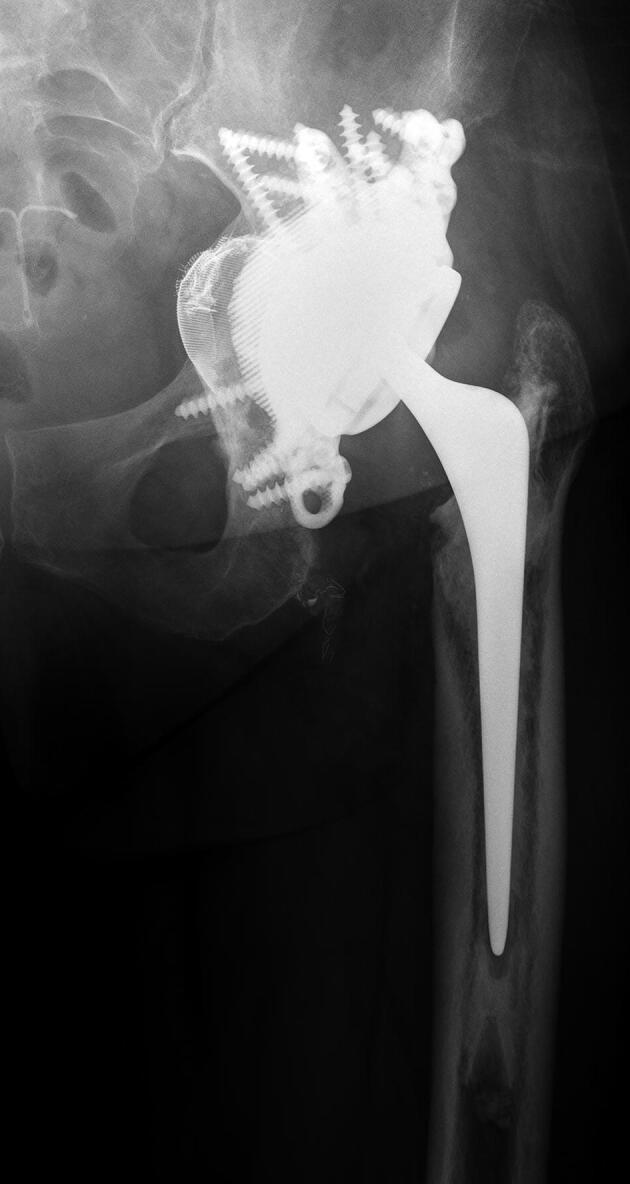

**Figure:**
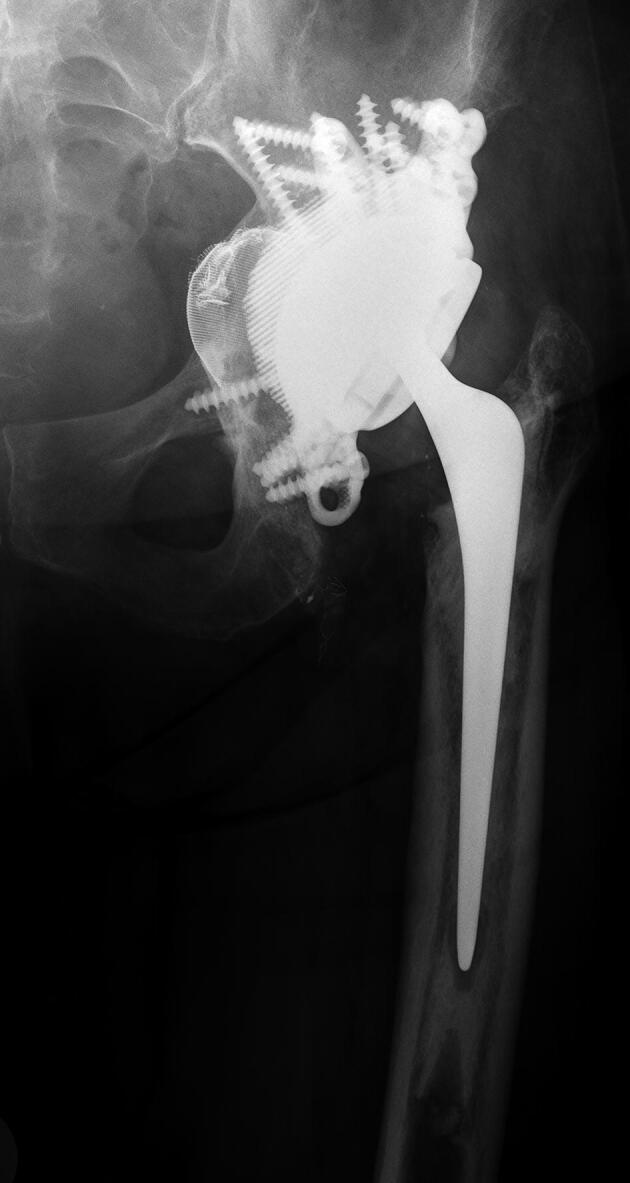

### Complications

In 49 cases the complication registration was complete. 8 cases had complications. Of these, 3 cases had re-explorations for persistent wound discharge (cases 6, 16, and 28), with collection of intraoperative cultures. In 1 of these cases (case 16) cultures were found to be positive, which was treated with 3 months of antibiotics. During the re-exploration of this same case 3 loose ischium screws and 1 loose pubis screw were exchanged. In 3 other cases a fracture of the pelvis (cases 2, 27, and 45) occurred, 2 postoperatively and 1 stress fracture after 6 months. These 3 cases were treated conservatively. The stress fracture evolved into a pseudoarthrosis; the other 2 fractures healed. At 3 weeks postoperatively, in another case a hip dislocated (case 3), which was treated conservatively with closed reduction and a brace and the hip did not dislocate again at the 2-year FU. This patient had ischiatic nerve irritation due to the dislocation. In the 8th case with complications, a general complication occurred, which involved a cerebrovascular accident directly postoperatively (case 26).

The rates of mOHS and complications were similar in patients with and without PD (Table 4).

**Table 4.** Clinical and patient-reported outcomes of hips with and without PD

	No PD (n = 33)	PD (n = 16)
mOHS preoperative mean	52 (24–69)	53 (25–60)
mOHS postoperative	28 (14–48)	32 (17–56)
Overall clinical complication rate	5	3
Dislocation rate	1	0

PD, pelvic discontinuity; mOHS, modified Oxford Hip Score.

## Discussion

To our knowledge this is the 1st prospective study on a large group of patients with this particular custom-made implant and in which pre- and postoperative patient-reported clinical outcome scores are compared (Colen et al. 2013, Baauw et al. 2017, Myncke et al. 2017). This study is the 2nd prospective case series, deBoer et al. (2007) being the 1st on the results of any CTAC for large acetabular defects. Furthermore, patient satisfaction is evaluated in more detail compared with most studies on CTACs and it is the 1st that reports on the clinical relevance of the improvement in patient-reported functioning over time (De Martino et al. 2019, Chiarlone et al. 2020).

In this study, all of the clinical patient-reported outcome scores improved over time, which is consistent with other studies on CTACs (De Martino et al. 2019, Chiarlone et al. 2020). The improvement in the mOHS between preoperatively and 2-year FU was also found to be clinically relevant.

When comparing our study with 2 recent review articles on CTACs, the revision rate, overall reoperation rate, and the complication rate were lower in our study (De Martino et al. 2019, Chiarlone et al. 2020). In particular, our low dislocation rate (1/49) is notable. Risks of a high dislocation rate in revision THA include multiple previous hip revisions (Kosashvili et al. 2011), abductor muscle deficiency, and severe acetabular bone loss (Faldini et al. 2018), all of which are often present in hips that are managed with a CTAC, the current study included. Another risk factor is the revision of only 1 component (Faldini et al. 2018), which was the case in 39/50 of the hip revisions in the current study. We believe that the low number of dislocations in our study is related to the preoperative planning of implant anteversion, with the use of either a dual mobility design or a constrained liner, in the case of abductor deficiency, in all of our cases (Faldini et al. 2018). This assumption is supported by 2 other studies on CTACs that reported no dislocations and either used a dual mobility cup in all cases (Colen et al. 2013) or a constrained liner in most of their cases (Berasi et al. 2015). To our knowledge, only 2 other studies have measured the accuracy of the placement of their custom-made implant (Weber et al. 2019, ­Zampelis and Flivik 2020). Both of them found similar good placement accuracy, as we have previously found (Baauw et al. 2016), and had 1 and 0 dislocations in 11 and 10 patients, highlighting the importance of accurate placement to diminish the dislocation rate.

Another notable finding in our study is the low deep infection rate, 1 of 49. Known risk factors for deep infections after total hip arthroplasty include an ASA score of 3 or higher, a longer duration of surgery (Urquhart et al. 2010) and a higher number of previous revisions (Kosashvili et al. 2011). In our patients the median (range) previous revisions were 2 (1–9) and 6 patients had an ASA classification of 3. However, the 1 patient with a deep infection (case 16) had an ASA classification of 2 and had 2 previous revisions. We did not report on the surgical time, but we assume this was relatively short compared with other hip revision surgeries because all operations were performed by 2 orthopedic surgeons and because of the precise preoperative planning. Other factors that might explain our low infection rate are the following measurements that are routinely done in all THA revisions in our clinic: preoperative infection workup with lab work and intra-articular aspiration, the routine use of antibiotics perioperatively for at least 24 hours, intraoperative betadine lavage and irrigation, and finally meticulous wound closure and low-suction wound dressing in patients with a BMI of over 30.

When comparing revisions with PD and without PD we found similar results. This is in line with findings of 2 recent review articles on the treatment of PD that have found CTACs to be a viable treatment option (Szczepanski et al. 2019, Malahias et al. 2020). In our study there were no mechanical failures and no dislocations and the overall complication rate was 3 out of 16 in cases with PD. These results are favorable, not only compared with other studies on CTACs for PD but also when compared with other treatment options for PD, including cup-cages, anti-protrusion-cages, acetabular shells with plates, and pelvic distraction techniques (Szczepanski et al. 2019, Malahias et al. 2020).

There are some limitations in this study. 1st, the relatively short FU of 24 months. The average FU was found to be 5 years (range 1–18) in previous studies on CTACs (De Martino et al. 2019, Chiarlone et al. 2020). We will continue to follow up our patients. Another limitation is the fact that we cannot comment on the migration of the implant, which is difficult to determine for this particular implant on conventional radiographs. Recently, Zampelis and Flivik (2020) have determined the migration of a similar implant, same cage but without an augment, at 1-year follow-up using CT scans. They found small measured migration values of less than 1 degree or 1 mm. To determine the secondary stability of these implants in the long run new CT-based migration research will be necessary.

In conclusion, this CTAC used in large acetabular defects with and without PD demonstrates a relevant improvement in patient-reported daily functioning, high patient-reported satisfaction, few complications and no re-revisions at 2-year FU.

## Supplementary data

Table 1 is available as supplementary data in the online version of this article, http://dx.doi.org/10.1080/17453674.2021.1885254
